# Classification of ^18^F-Flutemetamol scans in cognitively normal older adults using machine learning trained with neuropathology as ground truth

**DOI:** 10.1007/s00259-022-05808-7

**Published:** 2022-05-06

**Authors:** Mariska Reinartz, Emma Susanne Luckett, Jolien Schaeverbeke, Steffi De Meyer, Katarzyna Adamczuk, Dietmar Rudolf Thal, Koen Van Laere, Patrick Dupont, Rik Vandenberghe

**Affiliations:** 1 Laboratory for Cognitive Neurology, KU Leuven, Leuven, Belgium; 2 Alzheimer Research Centre KU Leuven, Leuven Brain Institute, Leuven, Belgium; 3 Bioclinica, Newark, CA 94560 USA; 4 Department of Pathology, UZ Leuven, Leuven, Belgium; 5 Laboratory of Neuropathology, KU Leuven, Leuven, Belgium; 6 Division of Nuclear Medicine, UZ Leuven, Leuven, Belgium; 7 Nuclear Medicine and Molecular Imaging, Department of Imaging and Pathology, KU Leuven, Leuven, Belgium; 8 Neurology Department, University Hospitals Leuven, Herestraat 49, 3000 Leuven, Belgium

**Keywords:** Positron emission tomography (PET), ^18^F-Flutemetamol, Alzheimer’s disease (AD), Neuropathology, Classification, Support vector machine (SVM)

## Abstract

**Purpose:**

End-of-life studies have validated the binary visual reads of ^18^F-labeled amyloid PET tracers as an accurate tool for the presence or absence of increased neuritic amyloid plaque density. In this study, the performance of a support vector machine (SVM)-based classifier will be tested against pathological ground truths and its performance determined in cognitively healthy older adults.

**Methods:**

We applied SVM with a linear kernel to an ^18^F-Flutemetamol end-of-life dataset to determine the regions with the highest feature weights in a data-driven manner and to compare between two different pathological ground truths: based on neuritic amyloid plaque density or on amyloid phases, respectively. We also trained and tested classifiers based on the 10% voxels with the highest amplitudes of feature weights for each of the two neuropathological ground truths. Next, we tested the classifiers’ diagnostic performance in the asymptomatic Alzheimer’s disease (AD) phase, a phase of interest for future drug development, in an independent dataset of cognitively intact older adults, the Flemish Prevent AD Cohort-KU Leuven (F-PACK). A regression analysis was conducted between the Centiloid (CL) value in a composite volume of interest (VOI), as index for amyloid load, and the distance to the hyperplane for each of the two classifiers, based on the two pathological ground truths. A receiver operating characteristic analysis was also performed to determine the CL threshold that optimally discriminates between neuritic amyloid plaque positivity versus negativity, or amyloid phase positivity versus negativity, within F-PACK.

**Results:**

The classifiers yielded adequate specificity and sensitivity within the end-of-life dataset (neuritic amyloid plaque density classifier: specificity of 90.2% and sensitivity of 83.7%; amyloid phase classifier: specificity of 98.4% and sensitivity of 84.0%). The regions with the highest feature weights corresponded to precuneus, caudate, anteromedial prefrontal, and also posterior inferior temporal and inferior parietal cortex. In the cognitively normal cohort, the correlation coefficient between CL and distance to the hyperplane was −0.66 for the classifier trained with neuritic amyloid plaque density, and −0.88 for the classifier trained with amyloid phases. This difference was significant. The optimal CL cut-off for discriminating positive versus negative scans was *CL* = 48–51 for the different classifiers (area under the curve (*AUC*) = 99.9%), except for the classifier trained with amyloid phases and based on the 10% voxels with highest feature weights. There the cut-off was *CL* = 26 (*AUC* = 99.5%), which closely matched the CL threshold for discriminating phases 0–2 from 3–5 based on the end-of-life dataset and the neuropathological ground truth.

**Discussion:**

Among a set of neuropathologically validated classifiers trained with end-of-life cases, transfer to a cognitively normal population works best for a classifier trained with amyloid phases and using only voxels with the highest amplitudes of feature weights.

**Supplementary Information:**

The online version contains supplementary material available at 10.1007/s00259-022-05808-7.

## Introduction

Key evidence for the validity of amyloid PET tracers comes from end-of-life studies [1–3]. In these studies, visual reads of scans based on PET tracers ^18^F-Florbetapir, ^18^F-Florbetaben, and ^18^F-Flutemetamol had a high diagnostic accuracy for predicting the presence of neuritic amyloid plaques. As an example, in the extended dataset from the pivotal ^18^F-Flutemetamol end-of-life study [2] based on 106 cases[4], sensitivity of ^18^F-Flutemetamol PET by majority read for increased neuritic plaque density was 91% and specificity was 90% [4, 5]. Comparable diagnostic accuracy was found for ^18^F-Florbetapir [1] and ^18^F-Florbetaben [3].

In these pivotal clinicopathological studies, the visual read was based on a set of prior ad hoc rules for discriminating positive vs negative scans. In a previous study, a support vector machine (SVM) with a linear kernel was trained with the ^18^F-Flutemetamol phase 2 data and compared to visual reads [6]. The classifier was able to replicate the visual reads with 100% concordance and revealed that the highest feature weights were localized to the striatum, precuneus, cingulate, and middle frontal gyrus. Training and testing a classifier for binary classification against a neuropathological ground truth may provide us with a more data-driven way of defining the most discriminative features, rather than an expert- or consensus-based definition based on visual read rules. Furthermore, in contrast to visual reads, SVM provides the distance to the hyperplane as a continuous measure of the level of certainty of the classifier. The hyperplane is the plane that separates the cases belonging to the two classes according to the SVM with a linear kernel. The distance to the hyperplane is a measure of the strength of evidence that the classifier has for putting a case in one or the other class. This continuous measure can then be correlated with a continuous neuropathological measure.

SVM can also be trained with a different ground truth to see whether its diagnostic performance against different neuropathological dimensions outperforms that of the a priori chosen measure of modified neuritic amyloid plaque density. Such an alternative neuropathological classification scheme is based on amyloid phases determined from Aβ immunochemistry [7, 8]. According to Thal et al. (2002), Aβ spreads hierarchically through the brain in 5 phases: from neocortical areas (Aβ phase 1), it spreads into allocortical regions including entorhinal cortex and hippocampus (phase 2), next to the basal ganglia, hypothalamus, and thalamus (phase 3), followed by the midbrain (phase 4), and eventually into the cerebellum and pons (phase 5) [7]. In the pivotal ^18^F-Flutemetamol end-of-life dataset (*n* = 68), all phase 0–2 cases were read as negative and 89% of phases 4–5 as positive, whereas 33% of the phase 3 cases were read as positive [9]. We grouped phases 0–2 and contrasted them with phases 3–5 and trained the classifier for this binary distinction. The grouping of cases with amyloid phases 0–2 as negative has been used before [10] and also corresponds to newly developed amyloid PET classification schemes that assume that phases 0–2 are not detectable in vivo with PET [11, 12].

Once the classifier has been trained and tested using post-mortem verification, and has proven to be accurate, it can readily be applied to different datasets without the need for laborious and time-intensive visual reads. This may be particularly useful for the detection of Alzheimer’s disease (AD) in the asymptomatic phase, since levels in the early disease stage may be equivocal and more difficult to read visually. Amyloid imaging has been instrumental in defining the asymptomatic phase of AD [13]. Whereas visual reads of AD dementia cases versus controls have a high inter-rater reliability, the binary categorization of amyloid scans obtained in cognitively intact cases is more difficult: in cognitively intact individuals, intermediary levels of amyloid burden exist that may be difficult to categorize. Hence, cognitively intact healthy individuals may be one of the potential use cases for application of a classifier that has been trained on neuropathologically verified cases that cover a range of neuritic amyloid plaque densities and amyloid phases. We examined how the performance of the two classifiers that have been trained based on the neuropathological ground truth of neuritic plaque density or amyloid phase, related to a commonly used measure for semi-quantitative assessment of amyloid burden, the Centiloid (CL) scale [14], in the Flemish Prevent AD Cohort-KU Leuven (F-PACK), a longitudinal observation cohort of older adults who are cognitively intact at study inclusion [15].

## Methods

### Description of the study cohorts

#### End-of-life study

**Fig. 1:**
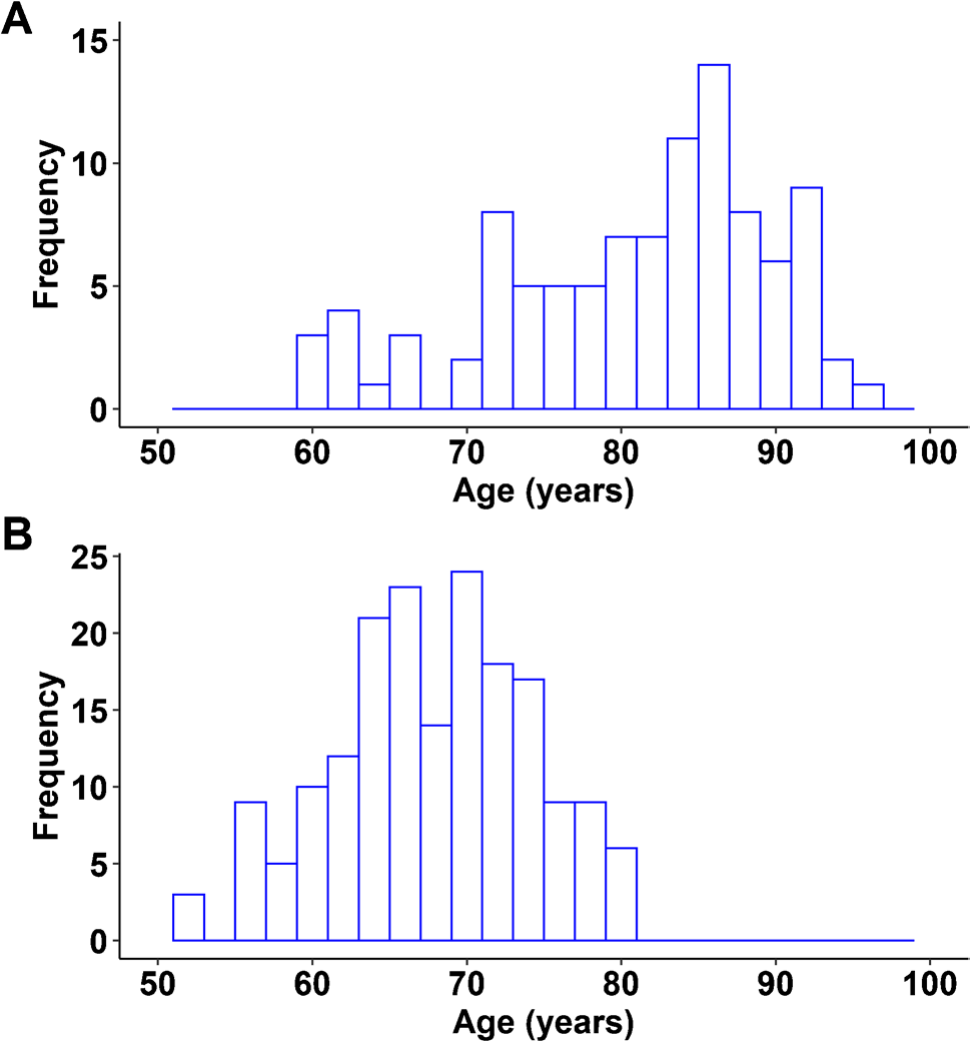
Age distribution. **A** Age distribution of the 101 cases of the end-of-life study. **B** Age distribution of the 180 subjects of the F-PACK cohort

A total of 101 cases (mean age 81.3 years, *SD* 8.9, range 60–96 years) from the phase 3 study [4] were included in the present study (Fig. 1A). The subjects had a cognitive status ranging from normal to advanced AD and, per protocol, had a life expectancy of 12 months or less. The mean scan-to-death interval was 222 days (*SD* 205.6, range 0–846 days).

From the initial 106 cases in this study [4], five cases had to be excluded a priori: two scans were not available in the dataset we had access to, one scan was excluded for a technical reason (the reconstructed image had voxel sizes which were > 4 mm), one scan was missing a part of the frontal lobe, and one scan had high extracerebral uptake in the nose region causing excessive frontal spill-in.

#### Flemish prevent AD cohort KU Leuven

F-PACK is a prospective longitudinal community-recruited cohort of 180 cognitively intact older adults (mean age 68.3 years, *SD* 6.4, range 52.4–80.9 years) (Fig. 1B), who all received an ^18^F-Flutemetamol PET scan at inclusion. This cohort was recruited between 2009 and 2015. A detailed description of the baseline characteristics of this cohort is available from Schaeverbeke et al. (2021) [15] and is also described in the supplementary materials.

## Image acquisition and analysis

### End-of-life study

In the GE067-026 trial, static ^18^F-Flutemetamol PET scans were acquired on PET/CT scanners from 90 to 100 min post injection in 94 cases and from 90 to 110 min in 7 cases, with an injected dose of 185–370 MBq (for details see Curtis et al. (2015) [2]). We used SPM12 running on MATLAB 2014b to process the images. To normalize the attenuation corrected PET scans to Montreal Neurological Imaging (MNI) space, a mean PET template was created from 62 SUVR ^18^F-Flutemetamol PET images from an independent in-house dataset [16]. These 62 PET images were normalized using their corresponding structural MRI scan. This template was then used to warp the individual PET scans from the end-of-life study to MNI space using a 12-parameter affine coregistration to the template followed by nonlinear deformations, whereby the deformations are defined by a linear combination of three dimensional discrete cosine transform basis functions [17]. An ^18^F-Flutemetamol Standardized Uptake Value Ratio (SUVR) image was created for each subject using the cerebellar grey matter (GM) as reference region obtained by intersecting the Automated Anatomical Labelling (AAL) atlas areas 91–108 [18] and the GM a priori map in MNI space (first volume of TMP.nii provided in SPM12) thresholded at 0.3. The SUVR images were smoothed with a 5 mm isotropic full-width half-maximum (FWHM) Gaussian 3D kernel.

### F-PACK study

For preprocessing of the PET scans from the F-PACK cohort, we made use of the individual’s structural MRIs. This differs from the PET-only procedure of the end-of-life data.

All F-PACK participants underwent ^18^F-Flutemetamol PET imaging on a 16-slice Biograph PET/CT scanner (Siemens, Erlangen, Germany). The tracer was injected as a bolus in an antecubital vein (mean activity 150 MBq, *SD* 5 MBq, range 134–162 MBq). Further details of the standard image acquisition procedure can be found in the Supplementary materials. Processing of ^18^F-Flutemetamol PET was done using SPM12 running on MATLAB R2014b as described in detail elsewhere [19, 20]. In short, a sumPET was created and the individual’s structural MRI scan was used for coregistration and normalization of the sumPET. A SUVR image was calculated using the subject-specific cerebellar grey matter as reference region obtained by intersecting the AAL areas 91–108 with the participants’ own GM map (thresholded at 0.3).

For reasons of comparability, the PET scans of the F-PACK cohort were also processed using the PET-only procedure as described above for the end-of-life data. For this a sumPET was created of the 90–110 min frames.

### Centiloid conversion regression formulas

We also expressed the overall amyloid PET load along the CL scale for both the MRI-assisted and the PET-only procedure. CL values were calculated as follows: Mean ^18^F-Flutemetamol SUVR values were calculated for a composite region, which consisted of five bilateral cortical regions (*SUVR*_comp_): frontal, parietal, anterior cingulate, posterior cingulate, and lateral temporal (the corresponding AAL areas are given in the Supplementary materials).

In the MRI assisted procedure, these regions were intersected with the participant-specific grey matter map, thresholded at 0.3. *SUVR*_comp_ values were then converted to CLs [14] using the following conversion formula: *CL* = 127.6 × *SUVR*_comp_ − 149 [21]. In order to obtain this conversion formula, the in-house processing procedure to obtain ^18^F-Flutemetamol *SUVR*_comp_ values was first calibrated against the standard Centiloid method [14], using an independent dataset as described before [21].

Since the end-of-life dataset did not contain MRI scans in the majority of the cases, we also calculated the CL conversion regression based on the PET-only procedure. CL values for the PET-only procedure were calculated as follows: Mean ^18^F-Flutemetamol SUVR values were calculated in *SUVR*_comp_. *SUVR*_comp_ values were then converted to CLs [14] using the following conversion formula: *CL* = 210.49 × *SUVR*_comp_ − 250.13. In order to obtain this conversion formula, the PET-only processing procedure to obtain ^18^F-Flutemetamol *SUVR*_comp_ values was first calibrated against the standard Centiloid method [14], using an independent dataset [22]. Further details are described in the Supplementary materials. For the PET images of the end-of-life study, CL values were calculated in the same way as for the PET scans of the F-PACK cohort processed using the PET-only procedure.

## Neuropathological ground truth

Neuropathological assessment data of the end-of-life study included Bielschowsky silver stain (BSS) Standard of Truth, Aβ phase [7], Consortium for the Establishment of a Registry for Alzheimer’s Disease (CERAD) score [23], Braak staging for tau pathology [24], the National Institute on Aging (NIA) and Ronald and Nancy Reagan Institute (NIA-RI) score [25], and the NIA-Alzheimer’s Association (NIA-AA) ABC score [8] for AD likelihood.

### Neuritic plaque density

In the phase 3 trial of ^18^F-Flutemetamol [2], the ground truth consisted of a binarized measure of the modified CERAD neuritic plaque density based on Bielschowsky silver staining. A binary categorization was based on neuritic plaque density as described in detail by Curtis et al. (2015) [2] and Ikonomovic et al. (2016) [4] and summarized in the supplementary materials. Normal cases had a neuritic plaque frequency of none or sparse (all regions had a mean neuritic plaque density ≤ 1.5) and the abnormal cases had a neuritic plaque frequency of moderate or frequent (at least one regional mean neuritic plaque density score > 1.5).

According to the CERAD neuritic plaque density Standard of Truth, the end-of-life dataset was composed of 29 negative and 72 positive cases (Table 1). These were then used to train the SVM.

**Table 1:** Demographics and neuropathology composition of the 101 cases of the end-of-life study

	Mean	*SD*	Range
Age (years)	81.3	8.9	60–96
Time-to-death (days)	222		0–846
^18^F-Flutemetamol SUVR	1.97	0.53	1.01–3.14
Neuropathology			
	BSS negative	BSS positive	
Total	29	72	
Aβ phase 0	7	0	
Aβ phase 1	10	0	
Aβ phase 2	5	0	
Aβ phase 3	5	14	
Aβ phase 4	1	21	
Aβ phase 5	1	44	

Abbreviations: *SUVR*, standardized uptake value ratio; *SD*, standard deviation; *BSS*, Bielschowsky silver stain

### Amyloid phase

Assessment of amyloid phases is described in previously published protocols [7, 8] and summarized in the supplementary materials. The distribution of the amyloid phases is listed in Table 1. All neuritic plaque density positive cases fell in amyloid phase 3–5, whereas 7 amyloid phase 3–5 cases were neuritic plaque density negative according to Bielschowsky silver staining.

### Centiloid threshold for positivity based on the end-of-life dataset

A receiver operating characteristic (ROC) analysis, using the R package *pROC* [26] (http://expasy.org/tools/pROC/), was applied to the CL values of the end-of-life dataset to determine the best CL threshold for neuritic plaque density (normal vs abnormal) and, separately, for Aβ phase (phase 0–2 vs 3–5). Area under the curve (*AUC*), and specificity and sensitivity at maximized Youden’s index were used as performance measures. The best CL threshold for both neuropathology scores was 28.9, with a specificity of 86.2% and a sensitivity of 73.6% for neuritic plaque density (*AUC* = 82.2%) and a specificity of 90.9% and a sensitivity of 69.6% for amyloid phases (*AUC* = 83.6%).

## Statistical analyses

Training of the SVM was performed in MATLAB (version 2014b) using the function fitcsvm with a linear kernel and default settings. An SVM with a linear kernel is defined by a hyperplane which subdivides the two classes. The distance to the hyperplane can be considered a quantitative measure of the strength of evidence that a case belongs to one or the other class. All standard statistical analyses were conducted with R statistical software version 4.0.3 (The R Foundation for Statistical Computing; cran.r-project.org). *P*-values were considered significant when meeting a two-tailed *α* threshold of 0.05.

### Primary analyses

A leave-one-out SVM was performed to discriminate the SUVR images of the end-of-life dataset in a binary manner based on normal versus abnormal neuritic plaque density as Standard of Truth (CERAD cut-off > 1.5), with accuracy, specificity, and sensitivity as outcome measures. To avoid bias, the number of cases in the positive class was equated to the number of normal cases (the negative class), which were the least abundant, resulting in 29 cases per class (Table 1). Selection of cases for the positive class was performed randomly 50 times, resulting in 50 classifiers. All scans except for one were used in the leave-one-out approach to train SVM. Every scan was left out once. The remaining scan was used as test set including the remaining cases of the biggest group. Specificity and sensitivity were determined. We also plotted the distance from the hyperplane against the neuritic plaque density. A negative distance corresponded to a positive case, and a positive distance corresponded to a negative case. This will be referred to as classifier ${}_{\mathrm{original}}^{BSS}$. A mean classifier was determined out of the 50 obtained classifiers.

**Fig. 2:**
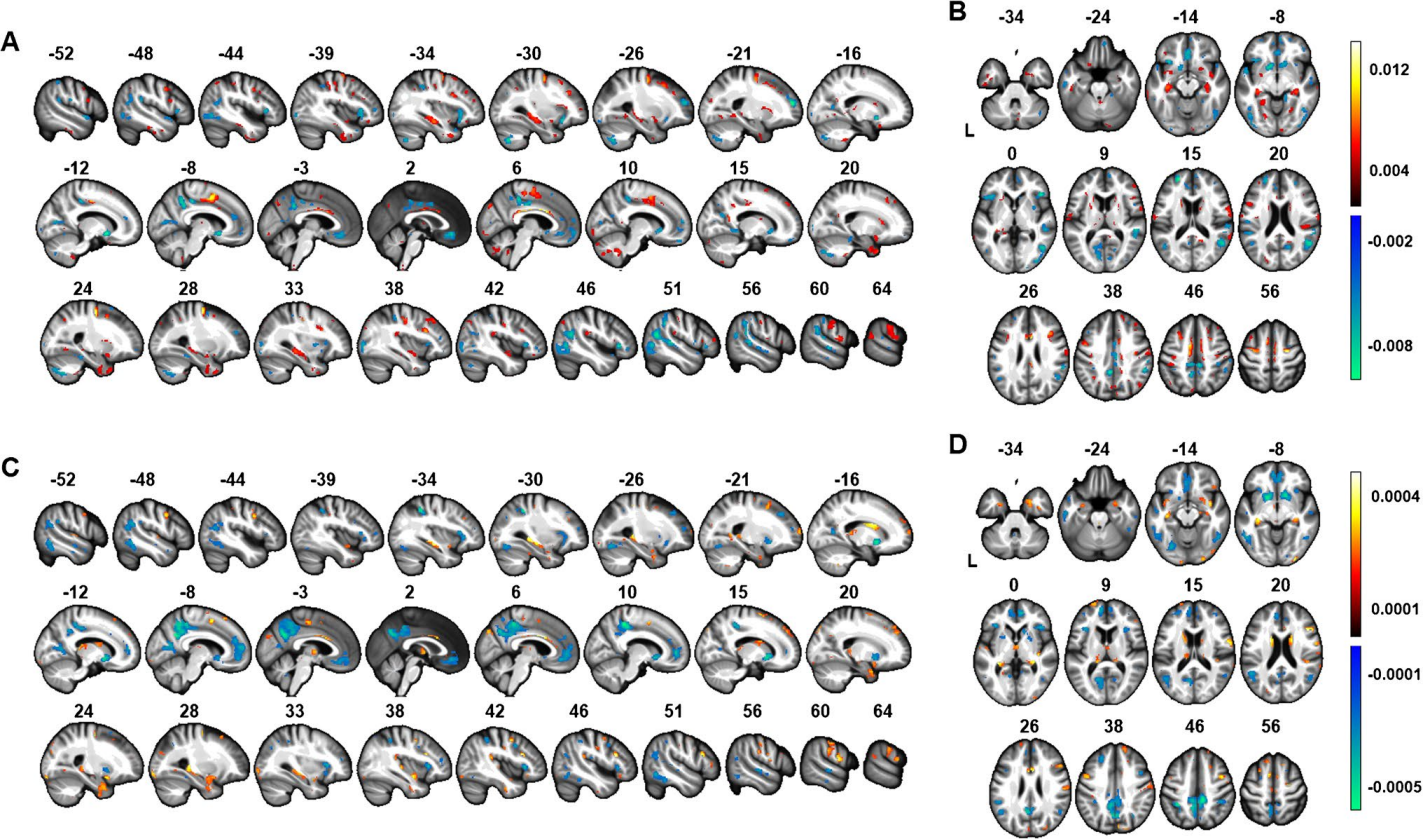
Distribution of the 10% voxels with the highest amplitudes of feature weights. **A**, **B** Distribution for the neuritic plaque density-based classifier visualized on **A** sagittal and **B** axial slices. **C**, **D** Distribution for the amyloid phase-based classifier visualized on **C** sagittal and **D** axial slices. In the sagittal slices (**A**, **C**), “negative” values correspond to the left hemisphere and “positive” values to the right hemisphere. The values in all panels refer to the slice location in MNI space in mm

The 10% voxels with the highest amplitudes of feature weights were extracted from this mean classifier for illustration in Fig. 2A. We also determined whether a classifier trained using only this selection of voxels was still sufficient for classification of the SUVR images. Therefore, we re-ran the analysis where only these voxels were used for classification of the SUVR images. This will be called the classifier ${}_{\mathrm{select}}^{BSS}$. For descriptive purposes, the specificity and sensitivity were determined and the distance from the hyperplane was plotted against the neuritic plaque density.

SVM was also trained to discriminate between Aβ phases 0–2 versus phases 3–5 applying the same leave-one-out approach, using 22 cases in each class since 22 cases had Aβ phases 0–2 and 79 cases had Aβ phases 3–5. This will be referred to as classifier ${}_{\mathrm{original}}^{A\beta }$. Again, a mean classifier was determined out of the 50 obtained classifiers.

The 10% voxels with the highest amplitudes of feature weights were extracted from this mean classifier for illustration in Fig. 2B. We also determined whether a classifier based on this subset of voxels allowed for accurate classification between Aβ phases 0–2 versus phases 3–5. Below, this will be referred to as classifier ${}_{\mathrm{select}}^{A\beta }$. Welch’s one-way ANOVA with no assumption of equal variances, with amyloid phase as between-subjects factor, was used to test if the distance to the hyperplane differed between amyloid phases. Then, pairwise *t* test with no assumption of equal variances was used to determine which amyloid phases differed significantly in distance to the hyperplane. *P-*values were adjusted using the Bonferroni multiple testing correction method.

Next, we evaluated how performance of the classifier related to the CL scale in asymptomatic older adults who participated in the F-PACK study. This was then compared to the CL threshold derived directly from the end-of-life dataset and to that determined in other studies [10]. This allowed us to evaluate which classifier transfers best from training with advanced cases (as in the end-of-life dataset), to application in asymptomatic cases (as in F-PACK). First, classifier ${}_{\mathrm{original}}^{BSS}$ and classifier ${}_{\mathrm{original}}^{A\beta }$ were applied for classification of the independent SUVR images in F-PACK (for both the SUVR images processed using the MRI-assisted procedure and the PET-only procedure). Second, we also used classifier ${}_{\mathrm{select}}^{BSS}$ and classifier ${}_{\mathrm{select}}^{A\beta }$ for classification in F-PACK. A simple linear regression was performed between the distance to the hyperplane and the CL value for both classifiers. To determine if these correlations differed significantly, the R package *cocor* [27] (http://comparingcorrelations.org) for comparison of two overlapping correlations based on dependent groups was used. The performance of all classifiers was also evaluated relative to the visual reads of the F-PACK cohort. We also evaluated which CL threshold optimally separates the cases considered pathological by the classifier from those considered normal. ROC analysis, using the R package *pROC* [26] (http://expasy.org/tools/pROC/), was applied to the CL values to determine which CL threshold best discriminates between classifier positive versus negative cases, for each of the classifiers. *AUC*, and specificity and sensitivity at maximized Youden’s index will be used as performance measures.

### Secondary analyses

As a secondary analysis, we evaluated the performance of two recently proposed PET amyloid staging schemes against the performance of the classifier. The PET amyloid staging scheme from Hanseeuw et al. (2018) is based on visual reads and consists of three stages: PET amyloid stage 0 (low cortical and low striatal PET signal), PET amyloid stage 1 (high cortical and low striatal PET signal), and PET amyloid stage 2 (high cortical and high striatal PET signal) [11]. The PET amyloid staging scheme from Thal et al. (2018) is based on visual reads plus semi-quantitative assessment of the cortex (SUVR_cortex_) and caudate nucleus (SUVR_caudatus_) with the pons as reference region. The Thal et al. (2018) staging scheme consists of four PET Aβ phase estimates: PET Aβ phase estimate 0 (without visible ^18^F-Flutemetamol retention), PET Aβ phase estimate 1 (cortical amyloid deposition; SUVR_cortex_ ≥ 0.5 and/or SUVR_caudatus_ ≥ 0.6), PET Aβ phase estimate 2 (majority reads also indicated amyloid positivity in striatum; SUVR_cortex_ ≥ 0.6 and/or SUVR_caudatus_ ≥ 0.7), and PET Aβ phase estimate 3 (SUVR_cortex_ ≥ 0.6 and/or SUVR_caudatus_ ≥ 1.0) [12]. The PET amyloid staging based on the two classification schemes was performed a priori by independent raters.

Using the end-of-life dataset, we examined how the distance to the hyperplane relates to these two PET amyloid staging schemes [11, 12]. Kruskal–Wallis rank sum test with amyloid phase as between-subjects factor was used to test if the distance to the hyperplane differed between PET amyloid phases (according to the Hanseeuw and Thal staging schemes, respectively). As the data were not normally distributed, the Mann–Whitney *U* test was used as post hoc analysis to determine which PET amyloid phases differed significantly in distance to the hyperplane. *P*-values were adjusted using the Bonferroni multiple testing correction method.

In addition, we examined how accurately the classifiers trained based on neuritic plaque density could classify according to amyloid phase and vice versa for the classifiers trained with amyloid phase.

## Results

### Primary analyses

#### End-of-life study

##### Neuritic plaque density as Standard of Truth

**Fig. 3:**
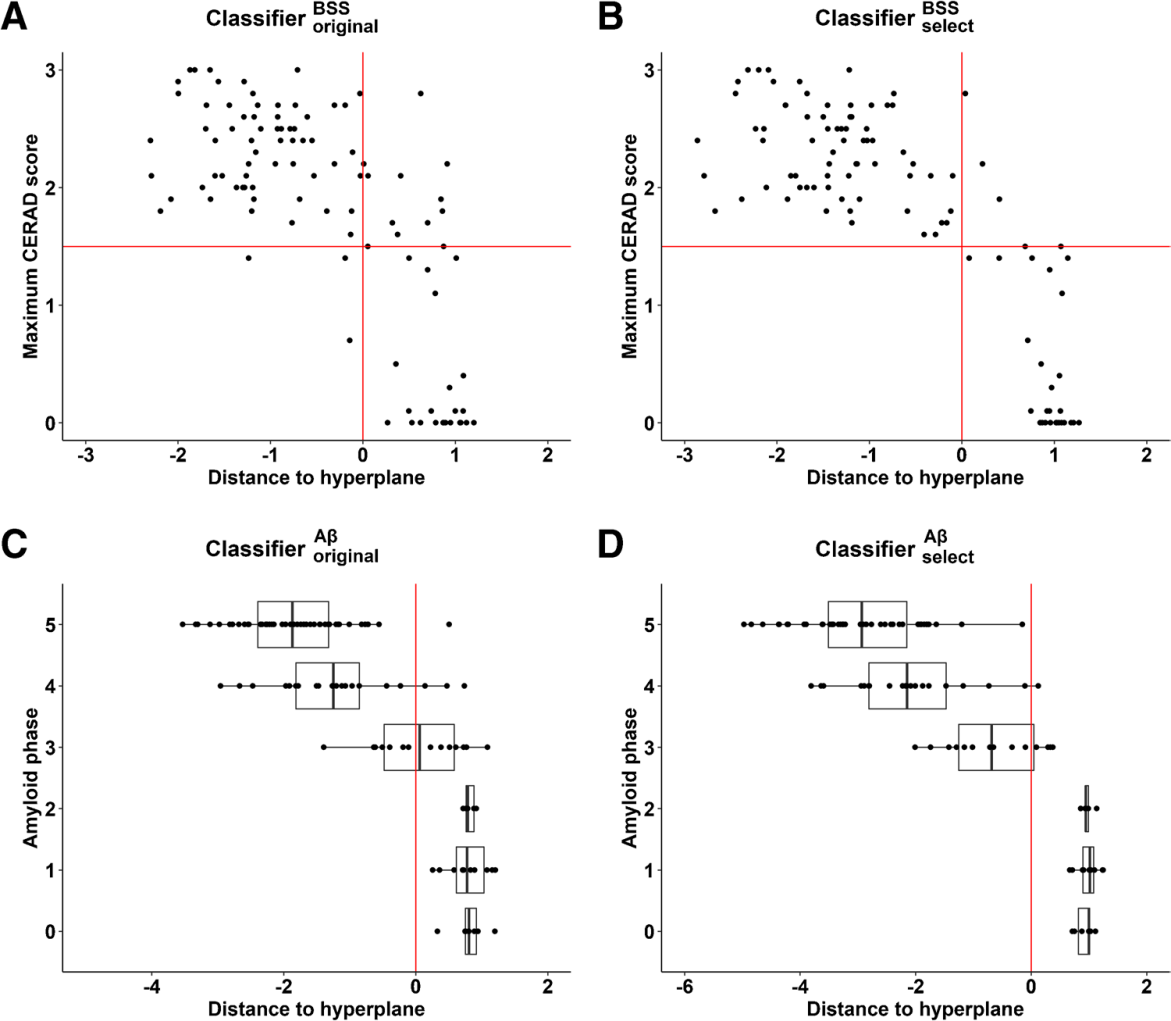
Classification of the 101 cases of the end-of-life study. **A** Regression plot between the maximum neuritic plaque density (CERAD) score and the distance to the hyperplane obtained from the classification of the 101 cases based on neuritic plaque density as Standard of Truth. Classification was based on classifier ${}_{\mathrm{original}}^{BSS}$. The maximum neuritic plaque density score is the score of the region with the highest score. A negative distance corresponds to a positive case; a positive distance corresponds to a negative case. **B** Regression plot between the maximum neuritic plaque density (CERAD) score and the distance to the hyperplane obtained from the classification of the 101 cases based on neuritic plaque density as Standard of Truth (Spearman *R* =  −0.73, slope =  −1.02, *P* < 2.2 × 10^–16^). Classification was based on classifier ${}_{\mathrm{select}}^{BSS}$. A negative distance corresponds to a positive case; a positive distance corresponds to a negative case. **C** Regression plot between the amyloid phase and the distance to the hyperplane obtained from the classification of the 101 cases based on amyloid phases 0–2 vs 3–5. Classification was based on classifier ${}_{\mathrm{original}}^{A\beta }$. A negative distance corresponds to a positive case; a positive distance corresponds to a negative case. **D** Regression plot between the amyloid phase and the distance to the hyperplane obtained from the classification of the 101 cases based on amyloid phases 0–2 vs 3–5 (Spearman *R* =  −0.81, slope =  −0.93, *P* < 2.2 × 10^–16^). Classification was based on classifier ${}_{\mathrm{select}}^{A\beta }$. A negative distance corresponds to a positive case. The boxplots visualize the minimum, first quartile, median, third quartile, and maximum distance per phase

An SVM was trained using the binary neuritic plaque density measure as Standard of Truth, as in the pivotal phase 3 study [2]. Compared to the Standard of Truth, the classifier had an accuracy of 87% (*SD* 3.6). The mean specificity was 90.2% (*SD* 4.3; 95% CI 89.0, 91.4; Fig. 3A): out of 29 Standard of Truth normal cases, three were classified as abnormal. Two were 87 years of age, one 92. The amyloid phase of these three cases was phase 3, 4, and 5 respectively. The CERAD plaque score was sparse in all three cases; the Braak stage was I, II, and IV, respectively. According to NIA-RI criteria [25], the AD likelihood was low in all three cases. When using the NIA-AA ABC score [8], two had a low AD likelihood and one had an intermediate AD likelihood. Thus, the false-positive cases had a relatively high amyloid load with relatively low neurofibrillary tangle deposition. The mean sensitivity was 83.7% (*SD* 4.9; 95% CI 82.4, 85.1; Fig. 3A) compared to the neuritic amyloid plaque density Standard of Truth: out of 72 abnormal cases, ten were classified as normal. Five of these cases were in amyloid phase 3, four were in amyloid phase 4, and one in amyloid phase 5. The CERAD plaque score was sparse in three of the cases, and moderate to frequent in the remainder. Three of the cases had Braak stage VI, two Braak stage IV, and the remainder had Braak stage III or lower. Hence, no clear pattern emerges why these cases were classified as normal.

To gain further insight in the relationship between the classification evidence strength for the classifier ${}_{\mathrm{original}}^{BSS}$ and the continuous neuropathological measures of neuritic plaque density, we performed a regression analysis between the distance to the hyperplane and neuritic plaque density. Distance to the hyperplane had a strong relationship with neuritic plaque density (Spearman *R* =  −0.66, slope =  −0.69, *P* < 5.7 × 10^–14^; Fig. 3A).

The 10% voxels with the highest amplitudes of feature weights are visualized in Fig. 2A. The pattern was relatively scattered. We trained and tested the classifier ${}_{\mathrm{select}}^{BSS}$ based on the values of this subset of voxels, and this classifier was applied to the F-PACK cohort (described below). For descriptive purposes, when classifier ${}_{\mathrm{select}}^{BSS}$ was applied to the SUVR images of the end-of-life dataset, specificity was 98.8% and sensitivity was 95% with an overall accuracy of 96.9% (Fig. 3B).

##### Amyloid phase as Standard of Truth

When SVM was trained using amyloid phases 0–2 versus phases 3–5 as classes, the classifier had a mean accuracy of 91.2% (*SD* 3.4). The mean specificity was 98.4% (*SD* 2.4; 95% CI 97.7, 99.0). The mean sensitivity was 84.0% (*SD* 5.8; 95% CI 82.4, 85.6). Among the 77 abnormal cases (phases 3–5), seven out of 14 phase 3 cases, three out of 24 phase 4 cases and one phase 5 case were classified as normal (Fig. 3C). The false-negative rate mainly in phase 3 cases is in line with what has been previously reported for visual reads [9].

Using linear regression, we examined the relation between the distance to the hyperplane and amyloid phase. Distance to the hyperplane had a strong relationship with amyloid phase (Spearman *R* =  −0.79, slope =  −0.97, *P* < 2.2 × 10^–16^; Fig. 3C).

Welch’s one-way ANOVA with amyloid phase as between-subjects factor was used to test if the distance to the hyperplane differed between amyloid phases. Post hoc comparison revealed that there was no difference in the distance to the hyperplane between phases 0, 1, and 2. The distance to the hyperplane differed significantly from phases 0, 1, and 2 versus each of the other phases (*P*_corrected_ ≤ 0.00061). Among the phases 3, 4, and 5, the distance also differed significantly (*P*_corrected_ ≤ 0.024; Fig. 3C). Phases 4 and 5 did not differ significantly after correction for multiple comparisons (*P*_corrected_ = 0.599).

The 10% voxels with the highest amplitudes of feature weights are visualized in Fig. 2B. We created a mask with the 10% voxels with the highest amplitudes of feature weights and trained and tested the classifier ${}_{\mathrm{select}}^{A\beta }$ with this subset. For descriptive purposes, classifier ${}_{\mathrm{select}}^{A\beta }$ was re-run based on the neuropathological dataset and Aβ phases 0–2 versus phases 3–5 as ground truth. Accuracy increased to 96.3%, specificity increased to 100%, and sensitivity to 92.6%. The distribution of the distance to the hyperplane per amyloid phase is visualized in Fig. 3D.

#### F-PACK cohort

The analyses of the end-of-life study provided us with four classifiers; these will be used to analyse the scans from the F-PACK cohort.

**Fig. 4:**
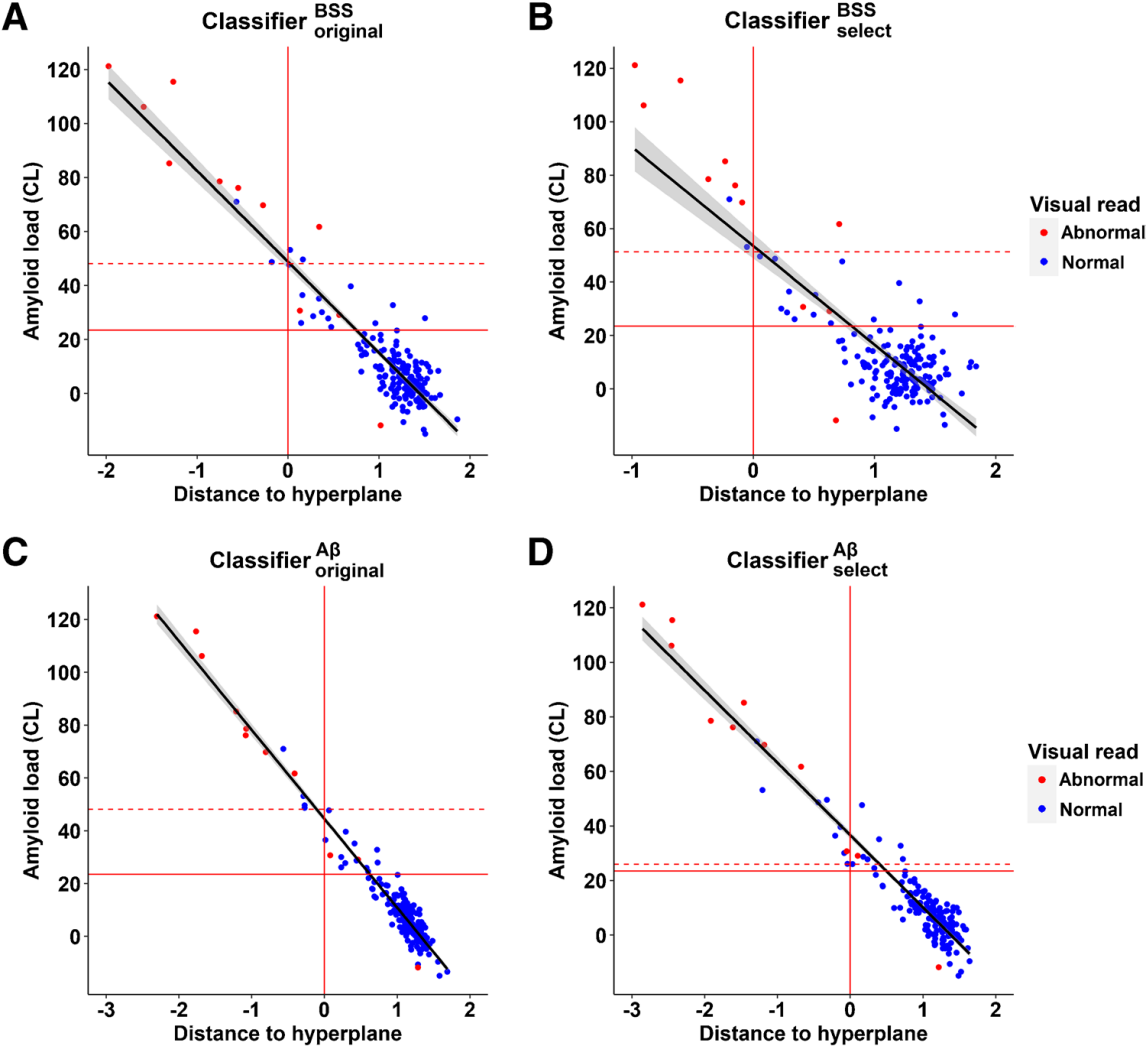
Classification of the F-PACK ^18^F-Flutemetamol SUVR images. **A** Regression plot between the Centiloid values and the distance to the hyperplane using the classifier based on neuritic plaque density (classifier ${}_{\mathrm{original}}^{BSS}$). The horizontal solid red line indicates the literature-based CL threshold of 23.5; the horizontal dashed red line indicates the CL threshold of 48.1 based on the ROC analysis. A negative distance corresponds to a positive case; a positive distance corresponds to a negative case. Abnormal visual reads are indicated in red and normal visual reads are indicated in blue. **B** Regression plot between the Centiloid values and the distance to the hyperplane using the classifier based on neuritic plaque density (classifier ${}_{\mathrm{select}}^{BSS}$). The horizontal solid red line indicates the literature-based CL threshold of 23.5; the horizontal dashed red line indicates the CL threshold of 51.3 based on the ROC analysis. A negative distance corresponds to a positive case; a positive distance corresponds to a negative case. Abnormal visual reads are indicated in red and normal visual reads are indicated in blue. **C** Regression plot between the Centiloid values and the distance to the hyperplane using the classifier based on amyloid phases 0–2 vs 3–5 (classifier ${}_{\mathrm{original}}^{A\beta }$). The horizontal solid red line indicates the literature-based CL threshold of 23.5; the horizontal dashed red line indicates the CL threshold of 48.1 based on the ROC analysis. A negative distance corresponds to a positive case. Abnormal visual reads are indicated in red and normal visual reads are indicated in blue. **D** Regression plot between the Centiloid values and the distance to the hyperplane using the classifier based on amyloid phases 0–2 vs 3–5 (classifier ${}_{\mathrm{select}}^{A\beta }$). The horizontal solid red line indicates the literature-based CL threshold of 23.5; the horizontal dashed red line indicates the CL threshold of 26.0 based on the ROC analysis. A negative distance corresponds to a positive case. Abnormal visual reads are indicated in red and normal visual reads are indicated in blue

A regression analysis was performed with the distance from the hyperplane as predictor and CL value as outcome variable in all 180 ^18^F-Flutemetamol F-PACK SUVR images. For classifier ${}_{\mathrm{original}}^{BSS}$, the correlation between the distance from the hyperplane and the CL value was −0.66, with a slope of −33.68 (*P* < 2.2 × 10^–16^; Fig. 4A) and for classifier ${}_{\mathrm{original}}^{A\beta }$, the correlation was −0.875, with a slope of −33.66 (*P* < 2.2 × 10^–16^; Fig. 4B). For classifier ${}_{\mathrm{select}}^{BSS}$, the correlation between the distance from the hyperplane and the CL value was −0.373, with a slope of −36.98 (*P* = 2.6 × 10^–7^; Fig. 4C). For classifier ${}_{\mathrm{select}}^{A\beta }$, the correlation was −0.814, with a slope of −26.5 (*P* < 2.2 × 10^–16^; Fig. 4D).

When comparing the performance of the classifiers to the visual reads, both classifier ${}_{\mathrm{original}}^{BSS}$ and classifier ${}_{\mathrm{select}}^{BSS}$ had a specificity of 98.8% and a sensitivity of only 63.6%. Classifier ${}_{\mathrm{original}}^{A\beta }$ had a specificity of 97.6% and a sensitivity of 72.7%, while the specificity and sensitivity of classifier ${}_{\mathrm{select}}^{A\beta }$ were 95.3% and 81.8%, respectively.

A regression analysis was also performed with the distance from the hyperplane as predictor and CL value as outcome variable in all 180 ^18^F-Flutemetamol F-PACK SUVR images, processed using the PET-only procedure. For classifier ${}_{\mathrm{original}}^{BSS}$, the correlation between the distance from the hyperplane and the CL value was −0.557 (*P* < 4.6 × 10^–16^) and for classifier ${}_{\mathrm{original}}^{A\beta }$, the correlation was −0.748 (*P* < 2.2 × 10^–16^). For classifier ${}_{\mathrm{select}}^{BSS}$, the correlation between the distance from the hyperplane and the CL value was −0.168 (*P* = 0.024). For classifier ${}_{\mathrm{select}}^{A\beta }$, the correlation was −0.616 (*P* < 2.2 × 10^–16^). These results are comparable to the results of the regression where the SUVR images were processed using the MRI-assisted procedure.

We also determined for each classifier the optimal CL threshold for separating cases classified as positive versus negative in F-PACK. When classifier ${}_{\mathrm{original}}^{BSS}$ was used as outcome for the ROC analysis, the CL threshold was 48.1 which resulted in a specificity of 98.25% and a sensitivity of 100%, both at the maximized Youden’s index, and an *AUC* of 99.8%. When classifier ${}_{\mathrm{original}}^{A\beta }$ was used as outcome, the CL threshold was 48.1, which resulted in a specificity of 100% and a sensitivity of 100%, both at the maximized Youden’s index, and an *AUC* of 100%. When classifier ${}_{\mathrm{select}}^{BSS}$ was used as outcome for the ROC analysis, the CL threshold was 51.3 which resulted in a specificity of 99.4% and a sensitivity of 100%, both at the maximized Youden’s index, and an *AUC* of 99.9%. On the other hand, when classifier ${}_{\mathrm{select}}^{A\beta }$ was used as outcome, the CL threshold was 26.0, which resulted in a specificity of 95.7% and a sensitivity of 100%, both at the maximized Youden’s index, and an *AUC* of 99.5%. In other words, for classifier ${}_{\mathrm{select}}^{A\beta }$, the CL threshold obtained was close to that obtained based on the ROC of the end-of-life dataset while for all other classifiers, the CL threshold was substantially higher. This confirms that the transfer of the classifier from an end-of-life dataset to an asymptomatic population was performing best for classifier ${}_{\mathrm{select}}^{A\beta }$.

### Secondary analyses: relation to PET amyloid staging schemes

**Fig. 5:**
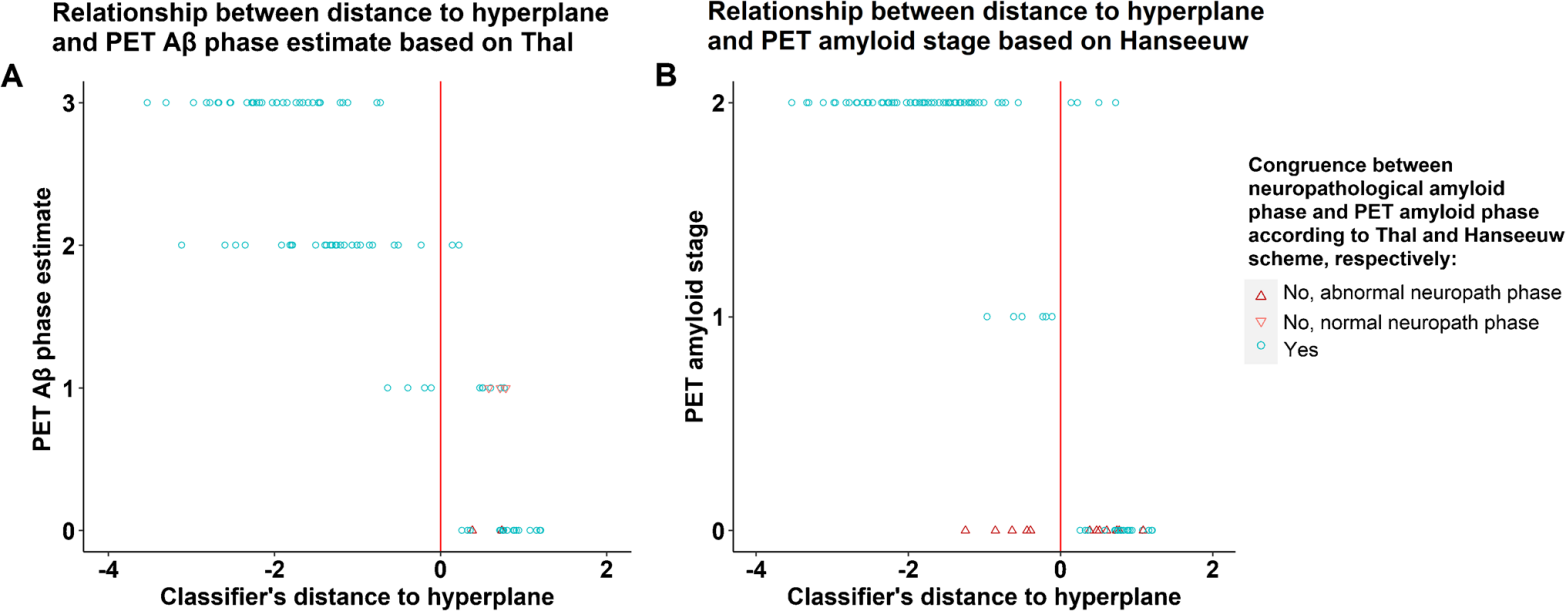
Relationship between the distance to the hyperplane and amyloid PET staging approaches. **A** The PET Aβ phase is based on Thal et al. (2018) [12]. The distance to the hyperplane is based on the classifier trained with amyloid phases 0–2 versus 3–5 (classifier ${}_{\mathrm{original}}^{A\beta }$). A negative distance corresponds to a positive case; a positive distance corresponds to a negative case. The PET Aβ phase estimates correspond to the underlying neuropathological amyloid phases [12] as follows: PET Aβ phase estimate 0 corresponds to neuropathological amyloid phases 0–2, PET Aβ phase estimate 1 to neuropathological phase 3, PET Aβ phase estimate 2 to neuropathological phase 4, and PET Aβ phase estimate 3 to neuropathological phase 5. The up-pointing triangles indicate that the neuropathological amyloid phase was abnormal. The down-pointing triangles indicate that the neuropathological amyloid phase was normal. **B** The PET amyloid stage is based on Hanseeuw et al. (2018) [11]. The distance to the hyperplane is based on the classifier trained with amyloid phases 0–2 versus 3–5 (classifier ${}_{\mathrm{original}}^{A\beta }$). A negative distance corresponds to a positive case; a positive distance corresponds to a negative case. The up-pointing triangles indicate that the neuropathological amyloid phase was abnormal

As a secondary analysis, we evaluated the performance of two recently proposed PET amyloid staging schemes against the performance of the classifier. One of these schemes is based on visual reads [11], the other on visual reads plus semi-quantitative assessment [12]. Using the end-of-life dataset, we examined how the distance to the hyperplane relates to these two PET amyloid staging schemes [11, 12]. Results are shown in Fig. 5.

As described before [12], the Thal et al. (2018) amyloid PET classification, which is based on SUVR in cortical and striatal regions [12], misclassified two neuropathological amyloid phase 3–5 cases as negative (i.e. placed them in the PET Aβ phase 0 category) and three neuropathological amyloid phase 0–2 cases as positive (i.e. placed them in the PET Aβ phase 1 category) (Fig. 5A). Of the 5 cases that were misclassified by the amyloid PET scheme [12], three were placed in the correct category by the classifier ${}_{\mathrm{original}}^{A\beta }$.

The Hanseeuw et al. (2018) amyloid PET classification, which is also based on amyloid deposition in cortical and striatal regions [11], misclassified twelve neuropathological amyloid phase 3–5 cases as negative. No neuropathological amyloid phase 0–2 cases were misclassified (Fig. 5B). A substantial number of cases (*n* = 12) that were placed in Hanseeuw phase 0 were false-negative. The classifier put 5 of these phase 3–5 cases in the pathological class, hence demonstrating superior performance.

Kruskal–Wallis rank sum test with amyloid PET stage as between-subjects factor was used to test if the distance to the hyperplane differed between amyloid PET phases. Post hoc comparison revealed a significant difference in the distance to the hyperplane per amyloid PET phase for the two amyloid PET classification schemes (Thal: *P*_corrected_ ≤ 0.017; Hanseeuw: *P*_corrected_ ≤ 0.0035).

We also tested whether training for a given neuropathological ground truth gave rise to a classifier that could also reliably classify for the alternative ground truth. When applying classifier ${}_{\mathrm{original}}^{BSS}$ to determine its ability to predict amyloid phase, the classifier’s accuracy was 92.0% with a specificity of 98.5% and a sensitivity of 85.4%. When classifier ${}_{\mathrm{select}}^{BSS}$ was used, the classifier’s accuracy was 94.0% with a specificity of 99.6% and a sensitivity of 88.4%. When applying classifier ${}_{\mathrm{original}}^{A\beta }$ to determine its ability to predict neuritic plaque score, the classifier’s accuracy was 87.3% with a specificity of 90.4% and a sensitivity of 84.3%. When classifier ${}_{\mathrm{select}}^{A\beta }$ was used, the classifier’s accuracy was 86.5% with a specificity of 87.6% and a sensitivity of 85.4%.

## Discussion

We trained a supervised machine learning classifier on the ^18^F-Flutemetamol end-of-life study and applied the classifier to an ^18^F-Flutemetamol dataset in a cohort of healthy cognitively intact older adults, the F-PACK cohort. In the F-PACK cohort, the Centiloid scale correlated more strongly with the performance of the amyloid phase-based classifier than with that of the neuritic plaque density-based classifier. Furthermore, the cut-off for discriminating positivity for neuritic plaque density was substantially higher than that for discriminating amyloid phases 3–5 from phases 0–2 based on classifier ${}_{\mathrm{select}}^{A\beta }$.

We used a leave-one-out approach for training and testing the classifier with the neuropathology as ground truth. The neuropathology ground truth is the major strength of this study. Ideally, the training and the test set are entirely independent. Given the limited number of autopsy cases available, a leave-one-out approach is the best approximation of this ideal. The case that is left out is independent of the cases on which the classifier is trained.

In the end-of-life dataset, when using neuritic plaque density as ground truth, mean specificity was practically the same (90%) as the median specificity reported by Ikonomovic et al. (2016) [4] for the visual reads. A pathologically negative case was only rarely classified as positive. This is in agreement with the approved indication of amyloid PET for ruling out AD. In the past, the false-positive cases have been attributed to amyloid in diffuse plaques and cerebral amyloid angiopathy and mismatched (sparse) neuritic plaque burden [4]. This is also a likely explanation for the classifier-based false-positives. The mean sensitivity of the classifier was numerically lower (83%) than the median sensitivity reported by Ikonomovic et al. (2016) [4] for visual reads (91%). In the past, the false-negatives have been attributed to advanced cortical atrophy and the absence of MRI availability, which may also account for the false-negatives in the classifier-based discrimination of the end-of-life dataset.

A classifier trained with amyloid phases 0–2 versus phases 3–5 in the end-of-life dataset performed in line with what one would expect based on the visual read studies [9]. Interestingly, the regions with the highest feature weights for discriminating phases 0–2 from phases 3–5 are not so much those that define amyloid phase 2 versus 3 neuropathologically (such as diencephalon and basal ganglia) but cortical regions. This indicates that the differentiation relies mainly on an overall increase in signal in key cortical areas and in the caudate nucleus due to increased concentration of Aβ aggregates/increased Aβ plaque load in the brain rather than the stepwise topographical expansion of Aβ plaque pathology as described by the amyloid phases [7]. In this context, it is essential to note that all aspects of Aβ pathology (its topographical expansion as described by the Aβ phases, the quantitative amounts of Aβ plaques/aggregates as measured by the Aβ plaque load or biochemically, and the maturation of Aβ aggregates) correlate closely with one another allowing a good estimation of all these parameters by amyloid PET [28]. The observation that a classifier trained on one neuropathological ground truth (neuritic plaque density or amyloid phase) could classify cases relatively accurately for the other ground truth (amyloid phase or neuritic plaque density, respectively) also testifies to this, at least when the dataset contains relatively advanced stages. As we will discuss further below, the interchangeability is less convincing for asymptomatic cases.

The sensitivity of the Centiloid (CL) method based on an ROC analysis of the end-of-life dataset was 73.6% for the neuritic amyloid plaque density and 69.6% for Thal amyloid phase. The classifier had a sensitivity of 83.7% and 84%, respectively. The sensitivity of the majority read based on the visual reads in the pivotal phase 3 study was 86%, with a confidence interval ranging from 73 to 95%, and the median of the sensitivity of the 5 readers was 88% (confidence interval 74%-96%) [2]. The sensitivity of the classifier (83.7%) falls within this range and the study demonstrates that the classifier performs similarly to the visual reads in that respect. Both the classifier and the visual reads take into account the distribution of the values across the entire scan rather than a single composite value and this may explain their similarity in performance and constitute an advantage compared to the CL method.

Recently, amyloid PET classification schemes have been proposed based on a combination of cortical and striatal amyloid levels or reads [11, 12]. The classifier correctly classified 3 out of 5 cases that the Thal et al. PET Aβ scheme misclassified. Five out of 12 cases misclassified by the Hanseeuw et al. PET amyloid scheme were correctly classified by the original classifier. A third 4-stage model of disease progression [29] exists. This scheme [29] has been developed principally through mathematical modelling of disease progression based on cross-sectional ^18^F-florbetapir amyloid PET scans in cognitively normal controls, mainly from the Alzheimer’s Disease Neuroimaging Initiative [29]. It has not been applied to the current end-of-life dataset as of yet and was not included in the current study for that reason.

Visual reads are based on a set of explicit ad hoc rules, hence the interest of a data-driven definition of the anatomical distribution of the most discriminative regions. The pattern obtained for the amyloid phase-based classifier confirmed the regions that are also considered critical for visual read classification: precuneus and posterior cingulate, head of the caudate, rostral anterior cingulate, and ventromedial prefrontal cortex. These are in line with a previous SVM paper with visual reads as comparison [6] and confirm the high feature weights of the head of the caudate nucleus as reported in that study and confirmed subsequently [11, 12, 30]. It also revealed some less commonly used regions, namely, the posterior inferotemporal cortex and the supramarginal gyrus. It is also worth noting that two of the three regions that define stage I in the Grothe et al. [29] staging scheme (basal temporal cortex, anterior cingulate, parietal operculum) are not among those with the highest feature weights.

More or less the same regions as for the amyloid phase-based classifier also had high feature weights for the neuritic plaque density-based classifier but the clusters for the latter classifier were more scattered and less confined. For the amyloid phase-based classifier, the visual appearance of the distribution of the highest feature weights corresponded better to regions commonly attended to for visual reads, than those of the neuritic plaque density-based classifier.

We applied the classifier to an independent ^18^F-Flutemetamol dataset obtained in 180 cognitively intact older adults. A classifier may be particularly useful in the asymptomatic stage of the disease, when a substantial portion of participants is situated at an intermediary level. From the SVM classifier, we derived the distance to the hyperplane for each SUVR image. This is a quantitative measure of the strength of evidence that a case belongs to one or the other class. It may be compared to the “level of confidence” of a visual read but is strictly objective. We used this measure of evidence strength to gain further insight in the link between the image classification and the continuous neuropathological measures underlying the binarized classification. When we extracted the distance from the hyperplane as a measure of classification likelihood, the distance from the amyloid phase-based classifier correlated more closely with the CL scale than when the same approach was taken for the neuritic plaque density-based classifier (*P* < 3.1 × 10^–15^). A closer match with amyloid phases compared to neuritic plaque density may be due to several reasons: amyloid phase takes into account both diffuse and neuritic plaques, and ^18^F-Flutemetamol has affinity for both types [31].

Two other findings indicated that the transfer to a cognitively normal population worked best for classifier ${}_{select}^{A\beta }$. The CL threshold for distinguishing amyloid 0–2 from 3–5 in the F-PACK cohort (*CL* = 26) closely corresponded to that obtained when determining a CL threshold directly from the end-of-life data (*CL* = 28.9). Second, classification based on classifier ${}_{\mathrm{select}}^{A\beta }$ corresponded best to the visual reads of the F-PACK cohort (spec 95.3%, sens 81.8%) compared to the other classifiers. The classifiers trained on the end-of-life data to classify based on neuritic plaque density, had a lower sensitivity, and were less able to detect asymptomatic cases with increased amyloid load. Classification using machine learning works best when the training data reflect the same distribution as the data on which the classifier is applied. When we train using the end-of-life data and apply the classifier on an asymptomatic cohort, this poses a challenge to the classifiers as the data on which the classifier was trained are distributed differently from that on which the classifier is applied. In particular, the end-of-life study will contain many more neuropathologically advanced cases than an asymptomatic cohort, so ready transferability cannot be assumed. Among the four classifiers, transferability was satisfactory mostly for classifier ${}_{\mathrm{select}}^{A\beta }$. The superiority of the amyloid phase-based classifier may relate to the affinity of the PET tracer not only for neuritic but also for diffuse amyloid plaques. The superior performance of the amyloid phase-based classifier in comparison to the neuritic plaque-based classifier may also have a neurobiological reason: in the course of Alzheimer’s disease, the timepoint when a case crosses from amyloid phase 2 to amyloid phase 3 occurs earlier than the timepoint when a case crosses from sparse neuritic plaque density to moderate neuritic plaque density [32]. Hence, in an asymptomatic group, a classifier trained to distinguish phases 0–2 from phases 3–5 may have a higher sensitivity for detecting positive cases than a classifier trained on distinguishing zero/sparse neuritic plaque density from moderate/severe density. The discrimination between phases 0–2 and phases 3–5 may be more suitable in an asymptomatic population than the discrimination between zero/sparse and moderate/severe plaque density since the latter distinction occurs later in the disease course than the distinction between the amyloid phases [32]. The selection of the 10% voxels with the highest amplitudes of feature weights clearly has a beneficial effect for classifier ${}_{\mathrm{select}}^{A\beta }$. This can be attributed to the fact that the voxels with the highest amplitudes of feature weights may also be those that are affected earliest in the disease course. By the selection procedure, we reduce the dimensionality of the image, removing voxels that in the asymptomatic phase of AD, may contribute noise. When classifier ${}_{\mathrm{select}}^{A\beta }$ was used, the CL threshold for distinguishing amyloid phases 0–2 versus 3–5 (*CL* = 26) was also very close to that reported by La Joie et al. (2019) (*CL* = 23.5) [10].

Some of the classifiers performed poorly on the F-PACK. One cannot simply assume good transfer of a classifier trained on end-of-life data for application in a cohort of a very different nature, who are cognitively normal. Among the four classifiers, classifier ${}_{\mathrm{select}}^{A\beta }$ performed best in the asymptomatic cohort, with a close correlation between the CL and the distance to the hyperplane, a close concordance with visual reads and a CL threshold for positivity that matched that based on the neuropathological dataset and that reported by La Joie et al. (2019). What would be the added value of using classifier ${}_{\mathrm{select}}^{A\beta }$ compared to visual reads or semiquantitative assessment and CL? Given the close correspondence with visual reads and CL, the added value does not lie so much in the classification of a single image as outcome would be highly concordant. Instead, given the increasing availability of large datasets, containing 100s or 1000s of amyloid PET scans, a validated classifier is an efficient method for processing and classifying images on a large scale. In addition to this scalability, a second advantage is the generalisability of its use across centres. The use of a classifier is an objective, reader-independent method that can be easily reproduced across centres provided the input to the classifier has been processed in a state-of-the-art manner. It is worth noting that here the performance of the classifier did not critically depend on the differences in acquisition method between the end-of-life study and the F-PACK study (different scanners and acquisition windows) and neither on the differences in image analysis procedure (PET-only or MRI-assisted). Hence, the advantage of the classifier is its efficient, automated use on large datasets, and its rater-independent objectivity in classifying cases.

## Limitations

Five amyloid phases exist, which we dichotomized into amyloid phases 0–2 and 3–5. This was motivated by the relatively low number of cases in each of the phases 0, 1, and 2, and by the observation that amyloid PET is unable to detect increases in retention in phases 0–2 [9, 12]. However, the dichotomized scale based on amyloid phases has not undergone extensive validation. Likewise, while the amyloid phase is a stepwise classification, the distance to the hyperplane that we derive from the classifier is a continuous estimate of how strong the evidence is in favour of one or the other binary class. Training a classifier to perform a more fine-grained distinction between stages requires more cases per class, in particular for the lower classes, than the current dataset offers.

## Conclusion

Automated classification using a neuropathologically validated SVM classifier with a linear kernel has value in the detection of an increased amyloid phase or neuritic plaque density in cognitively intact older adults. This study establishes the distance to the hyperplane as an informative and integrative measure with a strong relationship to measures of neuritic amyloid plaque density and, in particular, to amyloid phases.

## Supplementary Information

Below is the link to the electronic supplementary material.

Supplementary file1 (DOCX 45 KB)

## Data Availability

The data that support the findings of this study are available from the corresponding author upon reasonable request.
