# Supplementary material for: Classification of 18F-Flutemetamol scans in cognitively normal older adults using machine learning trained with neuropathology as ground truth
Source: Eur J Nucl Med Mol Imaging. 2022 May 6;49(11):3772–86. doi: 10.1007/s00259-022-05808-7 (PMC9399207; doi:10.1007/s00259-022-05808-7)
Supplement: Supplementary file 1 — Supplementary file1 (DOCX 45 KB) [file 259_2022_5808_MOESM1_ESM.docx]

**Reinartz et al. Classification of ^18^F-Flutemetamol scans in cognitively normal older adults using machine learning trained with neuropathology as ground truth**

**Supplementary material**

# **Supplementary methods**

## **Description of the study cohorts**

### Flemish Prevent AD Cohort KU Leuven

The F-PACK inclusion criteria were age between 50 and 80 years, a Clinical Dementia Rating global score of zero, a Mini Mental state Examination (MMSE) score ≥ 27, and the scores on a standard neuropsychological examination at baseline had to be within published norms. The exclusion criteria were a significant neurological or psychiatric history, a history of cancer, a contraindication for MRI (e.g. pacemaker, metal implants or severe claustrophobia), focal brain lesions on MRI or exposure to ionizing radiation for research procedures above 1 mSv within the year prior to the amyloid PET scan at baseline. The F-PACK participants were genetically stratified at inclusion according to a factorial design with two factors: APOE ε4 carrier status (carrier versus non-carrier) and Brain Derived Neurotrophic Factor (BDNF) codon 66 polymorphism (*met* carrier versus non-carrier). In each five-year bin, the four cells of the factorial matrix were equally represented, with matching for age, sex, and education level.

## **Image acquisition and analysis**

### F-PACK study

^18^F-Flutemetamol PET scan acquisition started 90 min after tracer injection and lasted for 30 min [1,2]. Prior to PET acquisition, a low-dose CT scan of the head (11 mAs) was performed for attenuation correction. Random and scatter correction were applied. Data were recorded in list mode and reconstructed into six five minute frames using ordered subsets expectation maximization (4 iterations x 16 subsets).

### Centiloid conversion regression formulas

The Automated Anatomical Labelling (AAL) atlas areas that were used for the composite region were: frontal (AAL areas 3-10, 13-16, 23-28), parietal (AAL 57-70), anterior cingulate (AAL 31-32), posterior cingulate (AAL 35-36) and lateral temporal (AAL 81-82, 85-90).

SUVR_comp_ values calculated between 90-110 min were converted to CL values. The level-1 analysis was replicated previously [3]. We calibrated the PET-only processing method for ^18^F-Flutemetamol SUVR (^FLUTE^SUVR) against the standard Centiloid method [4]. First, linear regression between ^11^C-PiB SUVRs (^PiB^SUVR) calculated with the standard CL method and ^FLUTE^SUVR calculated between 90-110 min post injection with our PET-only procedure yielded a slope *m* and an intercept *b* which were used to calculate ^PiB-Calc^SUVR values, so that:

$${}^{FLUTE}{SUVR=m \times{}^{PiB}{SUVR+b}}$$

$${}^{PiB-Calc}{SUVR}= \frac{{}^{FLUTE}{SUVR-b}}{m}$$

Next, CL values were calculated using the ^PiB-Calc^SUVR values and the 0- and 100-anchor points obtained through level-1 analysis, so that:

$${}^{FLUTE}{CL}=100 \times\frac{{}^{PiB-Calc}{SUVR}- {}^{PiB}{SU{VR}_{YC-0}}}{{}^{PiB}{SU{VR}_{AD-100}- {}^{PiB}{SU{VR}_{YC-0}}}}$$

Through linear regression between the SUVR values and the calculated CL values, the following conversion formula was obtained: $CL=210.49 \times{SUVR}_{90-110min}- 250.13$. Linear regression between ^PiB^SUVR values calculated with the standard CL method and ^FLUTE^SUVR values calculated with our own pipeline showed good agreement between the original method and our own method with R^2^ = 0.89, which is well above the recommended threshold of 0.7. The relative variance – defined by the ratio of the standard deviation (SD) of CL values calculated in the YCs of the reference cohort (15.91) to the SD of the CL values calculated in the YCs in the cohort described in the original CL publication (4.41) – was equal to 3.61.

As most scans from the end-of-life dataset were acquired from 90-100 min post injection and because this acquisition window was not available in the previous independent dataset, another conversion formula was determined by calibrating the PET-only method for 90-100 min post injection against the standard Centiloid method. For this, an independent in-house dataset [2] of cognitively intact older controls consisting of 50-70 min ^11^C-PiB scans and 90-120 min ^18^F-flutemetamol was used. We created ^18^F-flutemetamol sumPET images from 90-100 min post injection.

Through linear regression between the SUVR values and the calculated CL values, the following conversion formula was obtained: $CL=93.458 \times{SUVR}_{90-100min}- 94.766$. However, linear regression between ^PiB^SUVR values calculated with the standard CL method and ^FLUTE^SUVR values calculated with our own pipeline did not show good agreement between the original method and our own method with R^2^ = 0.47, which is below the recommended threshold of 0.7.

This last conversion formula was applied to calculate CL values between 90-100 min post injection for the end-of-life dataset. When these CL values were used for the ROC analysis, the best CL threshold for both neuropathology scores was 29.1, with a specificity of 84.0% and a sensitivity of 73.9% for neuritic plaque density (AUC = 80.8%) and a specificity of 89.5% and a sensitivity of 70.7% for amyloid phases (AUC = 82.6%).

The CL values of the end-of-life dataset did not significantly differ when applying the conversion formula for 90-100 min or for 90-110 min (*P* = 0.2). As the linear regression between ^PiB^SUVR values calculated with the standard CL method and ^FLUTE^SUVR values (between 90-100 min post injection) calculated with our own pipeline did not show good agreement (R^2^ < 0.7), we used the CL values based on the conversion formula for 90-110 min post injection for the ROC analysis in the main text.

## **Neuropathological ground truth**

### Neuritic plaque density

Assessment of neuritic plaque density is described in detail by Curtis et al. (2015) [5] and Ikonomovic et al. (2016) [6]. In summary, neuritic plaque density was assessed in eight predefined regions. Density was divided into four classes based on the number of plaques seen in the microscopic field in those regions: 0 = none (no plaques), 1 = sparse (1-5 plaques), 2 = moderate (6-19 plaques) and 3 = frequent (20+ plaques). Normal cases had a neuritic plaque frequency of none or sparse (all regions had a mean neuritic plaque density ≤ 1.5) and the abnormal cases had a neuritic plaque frequency of moderate or frequent (at least one regional mean neuritic plaque density > 1.5). For a ‘negative’ brain, all eight regions examined had to have a mean neuritic plaque density of lower or equal to 1.5, a ‘positive’ brain had a mean neuritic plaque density higher than 1.5 in at least one of the regions examined [5].

### Amyloid phase

Paraffin sections of 5 mm thickness from all blocks were stained with hematoxylin & eosin (H&E) and anti-Aβ antibodies (1:100, formic acid and heat pretreatment; anti-Aβ; 4G8, SIG-39220; Covance, USA). The phase of Aβ plaque pathology (Aβ phase) was assessed after screening of the Aβ-stained sections for plaque distribution according to previously published protocols [7,8].

# **References**

1. Vandenberghe R, Van Laere K, Ivanoiu A, Salmon E, Bastin C, Triau E, et al. 18F-flutemetamol amyloid imaging in Alzheimer disease and mild cognitive impairment: a phase 2 trial. Ann Neurol [Internet]. 2010;68:319–29. Available from: https://doi.org/10.1002/ana.22068

2. Adamczuk K, Schaeverbeke J, Nelissen N, Neyens V, Vandenbulcke M, Goffin K, et al. Amyloid imaging in cognitively normal older adults: comparison between (18)F-flutemetamol and (11)C-Pittsburgh compound B. Eur J Nucl Med Mol Imaging [Internet]. 2016;43:142–51. Available from: https://doi.org/10.1007/s00259-015-3156-9

3. De Meyer S, Schaeverbeke JM, Verberk IMW, Gille B, De Schaepdryver M, Luckett ES, et al. Comparison of ELISA- and SIMOA-based quantification of plasma Aβ ratios for early detection of cerebral amyloidosis. Alzheimers Res Ther [Internet]. 2020;12:162. Available from: https://doi.org/10.1186/s13195-020-00728-w

4. Klunk W, Koeppe R, Price J, Benzinger T, Devous MS, Jagust W, et al. The Centiloid Project: standardizing quantitative amyloid plaque estimation by PET. Alzheimers Dement [Internet]. 2015;11:1–4. Available from: https://doi.org/10.1016/j.jalz.2014.07.003

5. Curtis C, Gamez JE, Singh U, Sadowsky CH, Villena T, Sabbagh MN, et al. Phase 3 trial of flutemetamol labeled with radioactive fluorine 18 imaging and neuritic plaque density. JAMA Neurol [Internet]. 2015;72:287–94. Available from: https://doi.org/10.1001/jamaneurol.2014.4144

6. Ikonomovic MD, Buckley CJ, Heurling K, Sherwin P, Jones PA, Zanette M, et al. Post-mortem histopathology underlying β-amyloid PET imaging following flutemetamol F 18 injection. Acta Neuropathol Commun [Internet]. 2016;4:130. Available from: https://doi.org/10.1186/s40478-016-0399-z

7. Thal DR, Rüb U, Orantes M, Braak H. Phases of A beta-deposition in the human brain and its relevance for the development of AD. Neurology [Internet]. 2002;58:1791–800. Available from: https://doi.org/10.1212/wnl.58.12.1791

8. Hyman BT, Phelps CH, Beach TG, Bigio EH, Cairns NJ, Carrillo MC, et al. National Institute on Aging-Alzheimer’s Association guidelines for the neuropathologic assessment of Alzheimer’s disease. Alzheimers Dement [Internet]. 2012;8:1–13. Available from: https://doi.org/10.1016/j.jalz.2011.10.007
